# Association of TILs with clinical parameters, Recurrence Score® results, and prognosis in patients with early HER2-negative breast cancer (BC)—a translational analysis of the prospective WSG PlanB trial

**DOI:** 10.1186/s13058-020-01283-w

**Published:** 2020-05-14

**Authors:** Cornelia Kolberg-Liedtke, Gluz Oleg, Heinisch Fred, Feuerhake Friedrich, Kreipe Hans, Clemens Michael, Nuding Benno, Malter Wolfram, Reimer Toralf, Wuerstlein Rachel, Graeser Monika, Shak Steve, Nitz Ulrike, Kates Ronald, Christgen Matthias, Harbeck Nadia

**Affiliations:** 1 Gynäkologie mit Brustzentrum, Charité-Universitätsmedizin Berlin, Berlin, Germany; 2 West German Study Group, Mönchengladbach, Germany; 3 Ev. Hospital Bethesda, Breast Center Niederrhein, Moenchengladbach, Germany; 4 Institute of Pathology, Medical School Hannover, Hannover, Germany; 5 Department of Oncology, Clinics Mutterhaus der Borromaeerinnen, Trier, Germany; 6 Department of Gynecology and Obstetrics, Evangelical Hospital, Bergisch Gladbach, Germany; 7 Department of Gynaecology and Obstertrics, Faculty of Medicine and University Hospital Cologne, Cologne, Germany; 8 Department of Gynaecology and Obstetrics, University Clinics Rostock, Rostock, Germany; 9 Breast Center, Department of Gynecology and Obstetrics, University of Munich (LMU) and CCCLMU, Munich, Germany; 10 Genomic Health, Inc., Redwood City, CA USA

**Keywords:** Tumor-infiltrating lymphocytes (TILs), Adjuvant chemotherapy, Breast cancer, Disease-free survival, Hormone receptor status

## Abstract

**Background:**

The presence of tumor-infiltrating lymphocytes has been associated with prognosis and chemotherapy response, particularly in high-risk breast cancer subtypes. There is limited data so far as to (i) how tumor-infiltrating lymphocyte (TIL) measurements correlate with genomic measurements such as the Oncotype DX Recurrence Score® and (ii) whether the survival impact of TIL measurements varies according to different adjuvant systemic therapies.

**Methods:**

The WSG PlanB trial compared an anthracycline-free chemotherapy regimen (6x docetaxel/cyclophosphamide, TC) to an anthracycline-taxane sequence (4xEC followed by 4x docetaxel) in patients with intermediate-risk, HER2-negative early breast cancer (EBC). Patients with HR-positive HER2-negative EBC were further stratified to receive endocrine therapy alone vs. chemotherapy followed by endocrine therapy based on Recurrence Score results and nodal status. In this analysis, three independent observers quantified and categorized the presence of TILs among tumor samples from patients in PlanB. TIL measurements were correlated with clinical/pathological parameters and treatment outcome overall and according to the treatment arm.

**Results:**

Disease-free survival (DFS) rates were significantly better (*p* = .04) in HR-negative patients with high vs. intermediate TIL levels and were higher in low vs. intermediate TIL patients, however with borderline significance only (*p* = .06). There were no significant differences among TIL categories in HR+ patients. High RS categories, HR-negative status, and high KI67 were independently and significantly associated with high TIL categories. There was no significant impact of TIL category on DFS in patients treated by endocrine therapy only; however, in patients receiving chemotherapy, DFS in the intermediate TIL category was lower than that in the other categories.

**Conclusion:**

Although the presence of high TILs is associated with negative prognostic parameters such as high KI67 and HR-negative status among patients with HR-positive HER2-negative EBC, patients with high TILs show a favorable 5-year DFS in both HR-positive/HER2-negative and triple-negative breast cancer.

## Background

Evaluation of the presence and quantity of tumor-infiltrating lymphocytes (TILs) in breast cancer is increasingly regarded as an important tool for the estimation of prognosis and therapy response among patients with breast cancer. TILs are more frequently observed at higher levels in patients with triple-negative and HER2-positive than in those with estrogen receptor-positive, HER2-negative breast cancer [1–3].

Clinically, as the presence of TILs is increasingly understood as mirroring enhanced tumor immunogenicity, TIL analysis may provide a basis for early assessment of the efficacy of immunotherapy in breast cancer patients. Importantly, TILs have been associated with prognosis and with chemotherapy response in early breast cancer (EBC), particularly in the presence of other high-risk features [4, 5], and may therefore help to guide therapy decisions. Also, there is a solid body of evidence underscoring the importance of TILs to predict response to neoadjuvant chemotherapy [6] as well as to potentially be prognostic after neoadjuvant systemic therapy [7].

So far, there is limited data, as to whether the correlation between TILs and prognosis/prediction depends upon specific chemotherapy regimens and/or endocrine therapy alone. Furthermore, there are yet no clinical algorithms suggesting TIL assessment in breast cancer with the goal to alter treatment decisions in clinical routine. Therefore, we aimed to analyze the prognostic value of TILs in patients who received two distinct chemotherapy regimens as part of a randomized clinical trial. The randomized WSG PlanB trial enrolled 3198 patients with HER2-negative pN0/1 breast cancer. Recurrence Score® (RS) results were incorporated for risk stratification in hormone receptor-positive (HR) breast cancer; of these, 348 (low RS) patients received endocrine therapy alone; overall, 2449 patients were randomized to antacycline-free (6xTC) vs. standard anthracycline-taxane chemotherapy (4xEC-4xDoc) [8]. Since the Oncotype DX Recurrence Score was evaluated in a significant fraction of patients with HR-positive breast cancer after an early amendment of the trial, we were also able to correlate TIL measurements with RS results.

## Methods

### Patients

We analyzed tumor samples from patients recruited into the prospective phase 3 WSG PlanB trial [8] (Supplementary Figure 1). Briefly, from 2009 to 2011, PlanB enrolled 3198 patients (central tumor bank, *n* = 3073) using the Oncotype DX® Recurrence Score® (RS) to define a genomically low-risk subset of clinically high-risk pN0–1 EBC patients for treatment with adjuvant endocrine therapy (ET) alone. Following an early amendment, hormone receptor (HR)-positive, pN0–1 RS ≤ 11 patients were recommended to omit chemotherapy. Patients with RS ≥ 12, pN2–3, or HR-negative HER2-negative disease were randomized to anthracycline-free (6xTC, arm A) vs. anthracycline-containing chemotherapy (4xEC ➔ 4xDoc, arm B). Since a central tumor bank was prospectively established, tumor samples could be retrieved systematically.

Primary surgically removed tumor tissue was sent to the central pathology lab of Genomic Health Inc. (Redwood City, CA) for RS analysis. As previously reported, slide review, IHC, and fluorescence in situ hybridization (FISH) analysis were performed in an independent central laboratory (Institute of Pathology, Hannover Medical School, Hannover, Germany) [9]. Tumors were classified by local pathology as ER- or PR-positive if immunostaining was present in ≥ 1% of tumor nuclei. Centralized staining for Ki67 (clone 30–9 rabbit monoclonal; Ventana, Tucson, AZ) was performed using standard protocols. Ki67 was evaluated by one experienced breast pathologist in at least 100 tumor cells within the highest density area; the measurement was performed semi-quantitatively (in 5% increments) and quantitatively (in 1% increments).

### Tissue microarray analysis for TILs

Hematoxylin-eosin (HE)-stained full sections of formalin-fixed paraffin-embedded (FFPE) tumor blocks were carefully examined, and areas with representative invasive breast cancer tissue were macro-dissected by means of sampling two 1.4-mm (diameter) tumor core biopsies. Core biopsies were assembled in TMA acceptor blocks as described previously [10]. Whole slide sections of FFPE TMAs were deparaffinized and rehydrated conventionally and were stained in Mayer’s hemalaun (Merck, Darmstadt, Germany) and 0.5% eosin.

### Immunohistochemistry

Batch-based immunohistochemical staining was performed on whole slide sections (1 μm) of FFPE TMAs on a Benchmark Ultra (Ventana) automated stainer. The CC1 mild protocol (Ventana) was used for antigen retrieval and the monoclonal anti-CD45 antibody (clone: 2B11+PD7/26, Dako, Glostrup, Denmark) for immunodetection. Development of the immune reaction was achieved with the ultraView DAB kit (Ventana). For counterstaining, sections were incubated in modified Gill’s hematoxylin (48%, Ventana, ready-to-use) and 0.1 M Li_2_CO_3_/0.5 M Na_2_CO_3_ (bluing reagent, Ventana, ready-to-use) for 8 min and 4 min, respectively.

### Analysis of TILs

Stromal TILs were evaluated by a pathologist using a two-observer approach. Three independent observers evaluated digital sections on HE staining as previously suggested [11]. After the initial evaluation, one independent observer re-evaluated digital whole slide image (WSI) sections on HE staining. HE-stained slides were used for primary analysis. A third evaluator cross-checked plausibility considering the previous scoring, using additional information such as CD45 and CK5/14 staining to evaluate tumor composition. In case of heterogeneous spatial distribution, results were averaged. “Hot spots” were generally excluded from the analysis. Among patients with multiple tumor samples (due to multi-centricity, *n* = 40), the tumor site with the highest TIL count was selected for further analysis. “TIL counts” were then categorized into three “TIL categories” of “low TILs” (≤ 10% stromal TILs), “intermediate TILs” (> 10 to ≤ 50% TILs), and “high TILs” (> 50% TILs).

Overall, guidelines of the International TILs Working Group 2014 were followed to assess TILs. The cutoff of 50% is in accordance with the Recommendations of the International TILs Working Group 2014 (to distinguish a subgroup of lymphocyte-predominant breast cancer) [11]. The cutoff of 10% was chosen to distinguish a subgroup with low to no stromal tumor-infiltrating lymphocytes (sTILs) vs. a subgroup with some sTILs. A binary variable “sTIL status” (“high” vs. “intermediate or low”) was also coded, representing the subgroup of “lymphocyte-predominant breast cancer.”

### Statistical analysis

Spearman correlations of sTIL categories with clinical/pathological parameters (including central Ki67 expression, quantitative ER measurements, nodal involvement, and RS) were computed. Logistic regression was also used to quantify the impact of these factors on sTIL status (“high” vs. “intermediate and low”). The prognostic impact of (fractionally ranked) sTIL categories on disease-free survival (DFS) was estimated by Kaplan-Meier analysis and tested using log-rank statistics. DFS was defined as breast cancer recurrence, secondary cancer event, or death of any cause. No adjustment was made for multiple comparisons. Interobserver variability was characterized by Spearman correlations between sTIL values of samples with two available independent measurements and by Kruskal’s *gamma* and concordant fractions for ordinal categories.

## Results

### Study population

Two thousand nine hundred ninety-three patients of the PlanB trial had sTIL measurements available, for whom 2517 had valid follow-up (60 months). A consort diagram is given in Supplementary Figure 2. DFS in the sTIL population was very similar to that of the population as a whole and to DFS in the group with no sTIL measurements.

### Correlations of sTILs with clinical/pathological parameters

Table 1 lists patient characteristics of patients with available sTILS in association with clinical/pathological parameters. Of note, our analyses showed a significant association between sTIL measurements and HR status, Ki67 categories, and Recurrence Score categories.

**Table 1:** Patient characteristics according to sTIL categories. sTIL categories were defined as “low sTILs” (0–10% sTILs), “intermediate sTILs” (11–50% sTILs), and “high sTILs” (51–100% sTILs)

	All	Low sTILs (valid %)	Intermediate sTILs (valid %)	High sTILs (valid %)	***p*** value, chi-square
**Tumor stage**					
**pT1**	1499	1232 (82.2)	218 (14.5)	49 (3.3)	.760
**pT22**	1088	885 (81.5)	163 (15.0)	38 (3.5)	
**pT3**	89	75 (84.3)	13 (14.6)	1 (1.1)	
**pT4**	17	16 (94.1)	1 (5.9)	0 (0.0)	
**Nodal status**					
**pN0**	1843	1499 (81.3)	274 (14.9)	70 (3.8)	.021
**pN1**	978	829 (84.8)	130 (13.3)	19 (1.9)	
**pN2–3**	172	143 (831)	27 (15.7)	2 (1.2)	
**ER (local)**					
**Negative**	445	256(57.5)	143 (32.1)	46 (10.3)	< .001
**Positive**	2246	1952 (86.9)	252 (11.2)	42 (1.9)	
**PR local**					
**Negative**	625	399 (63.8)	172 (27.5)	54 (8.6)	< .001
**Positive**	2066	1809 (87.6)	223 (10.8)	34 (1.6)	
**Local HR status**					
**Negative**	419	236 (56.3)	138 (32.9)	45 (10.7)	< .001
**Positive**	2574	2235 (86.8)	293 (11.4)	46 (1.8)	
**Age at registration**					
**≤ 50**	984	794 (80.7)	163 (16, 6)	27 (2.7)	.055
**> 50**	2009	1677 (83.5)	268 (13.3)	64 (3.2)	
**Ki67 expression**					
**1–15**	1040	959 (92.2)	76 (7.3)	5 (0.5)	< .001
**15–34**	1383	1113 (80.5)	223 (16.1)	47 (3.4)	
**35–100**	305	159 (52.1)	112 (36.7)	34 (11.1)	
**Recurrence Score**					
**Low (0–11)**	449	423 (94.2)	25 (5.6)	1 (0.2)	< .001
**Intermediate (12–25)**	1509	1345 (89.1)	146 (9.7)	18 (1.2)	
**High (26–99)**	546	407 (74.5)	112 (20.5)	27 (4.9)	

### Correlations of sTIL categories with clinical/pathological parameters in HR-positive and triple-negative breast cancers

Given the strong association between sTIL categories and HR status, associations with clinical/pathological variables were calculated for patients with HR-positive and HR-negative status separately (Supplementary Table 1). Since recurrence scores were rarely available for patients with HR-negative tumors, we estimated a predictive multivariate model to infer “high sTILs” (> 50%) by logistic regression using just the variables HR status (odds ratio (OR) 0.35; *p* value < .001) and Ki67 expression (OR 1.59 *p* < .001), which thus were independently and significantly associated with sTIL status. To assess the association between HR status, sTILs, and KI67 expression further, we built a prediction model that had an AUC of about .80 (.76–.85) in the cohort.

### Correlations of sTILs with DFS in HR-positive vs. triple-negative breast cancers

**Fig. 1:**
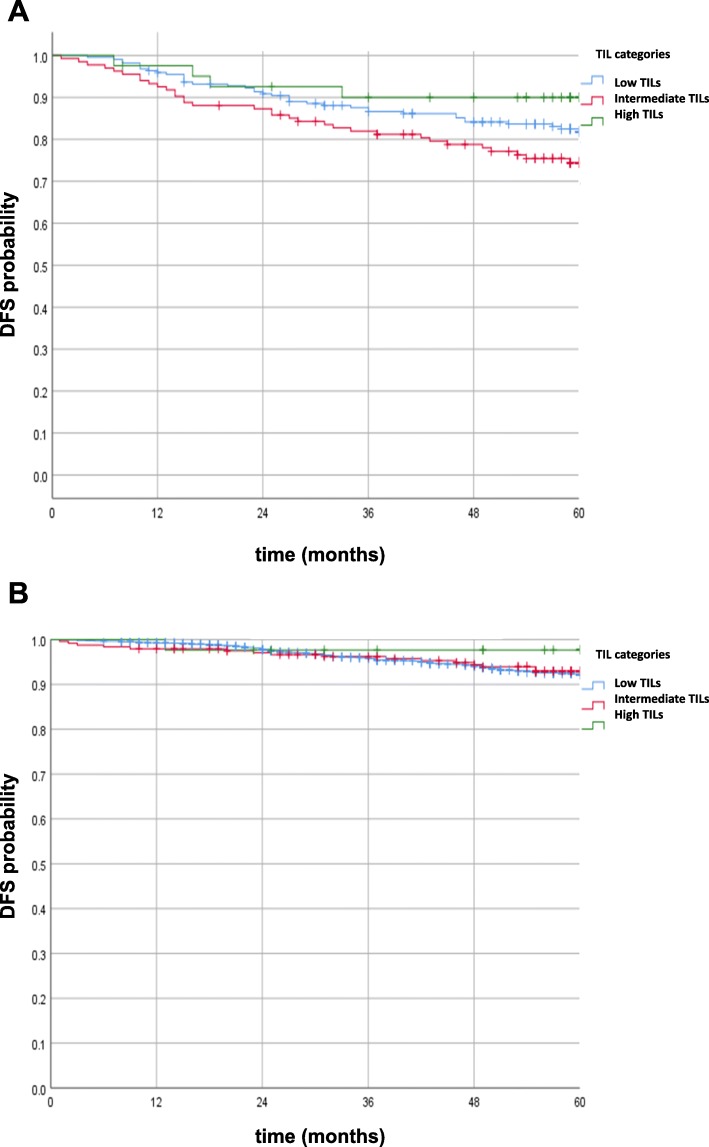
**a** Kaplan-Meier analysis of DFS according to sTIL categories (HR negative). **b** Kaplan-Meier analysis of DFS according to sTIL categories (HR positive)

We observed a significant association between sTIL categories and disease-free survival after stratification for HR status: among patients with HR-negative tumors and high vs. intermediate sTIL levels, DFS rates were significantly better (*p* = .04) and were higher (borderline significance, *p* = .06) in low vs. intermediate sTIL levels. We observed no significant difference among sTIL categories among patients with HR-positive tumors (Fig. 1a, b). Although numbers were small, DFS among patients with HR-positive disease and high sTILs was excellent.

### Association of sTIL expression and treatment arms

**Fig. 2:**
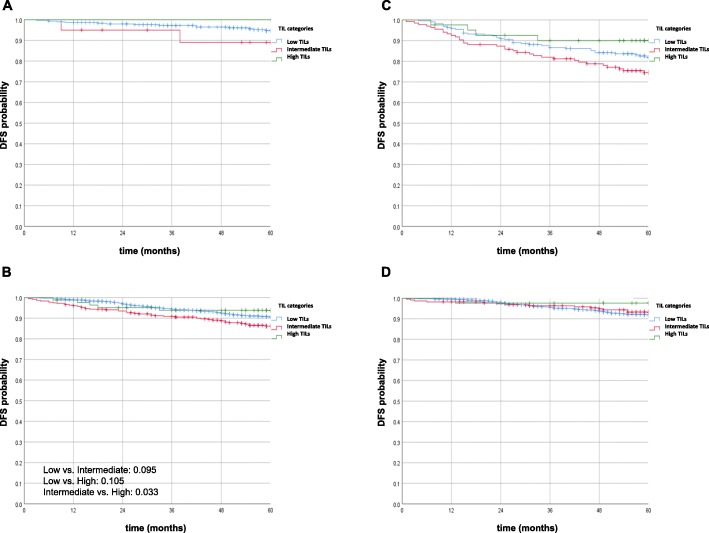
Kaplan-Meier analysis of DFS according to sTILs. **a** Among patients with RS ≤ 11 and endocrine therapy alone (no Kaplan-Meier analysis was performed to low number of patients/events). **b** Among patients treated with chemotherapy (irrespective of HR status). **c** Among patients with HR-negative tumors treated with chemotherapy. **d** Among patients with HR-positive tumors (and RS values of 12–99 treated with chemotherapy)

Figure 2 illustrates the impact of sTIL categories on DFS by chemotherapy treatment status and HR status. Whereas there was no significant impact of sTIL category on DFS in patients treated by endocrine therapy only (Fig. 2a, RS ≤ 11), in patients receiving chemotherapy, DFS in the intermediate sTIL category was lower than that in the other categories (Fig. 2b): the difference was significant compared to the low-sTIL group (*p* = .017) and borderline significant compared to the high-sTIL group (*p* = .07). Figure 2 c and d show that these differences are primarily attributable to the HR-negative subgroup. Overall, Kaplan-Meier analysis revealed no significant association between 5-year DFS and sTIL category according to chemotherapy treatment arm (arm A vs. arm B).

### Analysis of interobserver variation

The Spearman correlation between sTIL values of samples with two available independent measurements was 0.66 (Supplementary Figure 3). This correlation translates into a nearly ideal association (*gamma* = 0.944, concordance = 87.6%) in terms of the categories low, medium, and high.

## Discussion

The presence of sTILs in breast cancer tissue indicates that breast cancer has immunogenic properties, since the presence of sTILs may mirror the tumor’s ability to establish an adaptive immune response to the tumor cells. Clinically, this translates into a significant association between the presence of sTILs and both breast cancer prognosis [12–14] and response to chemotherapy [6, 15].

Herein, we present the results of a translational analysis of sTILs using tumor samples from the WSG PlanB trial, which compared an anthracycline-free chemotherapy regimen (6 x docetaxel/cyclophosphamide, TC) to an anthracycline-taxane sequence (4xEC ➔ 4xDoc) among patients with HER2-negative EBC. In summary, the presence of stromal sTILs was moderately associated with clinical features of high-risk breast cancer (including RS) in this dataset.

Our results are in line with previous analyses suggesting an association between high sTIL scores and prognosis among patients with triple-negative breast cancer. For instance, Carbognin et al. analyzed several adjuvant clinical trials and suggested a survival benefit for patients with triple-negative breast cancer in case of high sTILs (*p* < .0001) [16]. Furthermore, in a large pooled analysis, Denkert et al. confirmed a favorable effect of a high sTIL count among patients with triple-negative breast cancer. In univariable analysis, even a 10% increase in sTILs was associated with a significant increase in disease-free survival among patients with triple-negative disease (hazard ratio 0.93 (95% CI 0.87–0.98), *p* = 0.011) [6].

In Kaplan-Meier analysis, we observed a prognostic advantage of patients with high compared to intermediate sTILs in patients with HR-negative tumors (*p* = .04) and low vs. intermediate sTIL levels (borderline significance, *p* = .06). However, no significant difference among sTIL categories among patients with HR-positive tumors was observed. Furthermore, high sTIL categories were associated with high Ki67 expression and HR-negative status. The latter are established unfavorable prognostic but favorable predictive parameters regarding the benefit of chemotherapy [17].

Furthermore, we found a significant positive association between low sTIL categories and low RS measurements. Overall, there is yet limited data as to how sTIL measurements correlate with genomic predictors of patient prognosis. Dieci et al. analyzed the association between tumor-related and immune-related diversity of HER2-positive disease on the response to neoadjuvant chemotherapy plus anti-HER2 agents. They found that both tumor-related and immune-related features seemed to be associated with pCR after neoadjuvant chemotherapy plus anti-HER2 agents. However, immune signatures showed a more robust association with rates of pathological complete response than sTILs [18]. In a retrospective cohort analysis, Ahn and colleagues found a significant but weak correlation between stromal TIL levels and the RS in HR-positive breast cancer samples. In their analysis, the mean RS was found to be highest in high sTIL tumors (26.2 ± 8.2) vs. low and intermediate sTIL tumors (17.8 ± 10.7 and 19.4 ± 8.7, respectively, *p* = 0.014). In multivariate analysis, high RS could not be demonstrated to be an independent factor corresponding to high sTIL levels [19]. Notably, our analysis represents the first prospective-retrospective analysis of TIL categories in the context of the Recurrence Score. We found TILs to be significantly and strongly associated with RS measurements. A multivariate model for high TILs (> 50) using the variables HR status and Ki67 had AUC of about .80 (.76–.85) within our dataset. This degree of association suggests that although sTILs are associated with other prognostic variables, their impact on DFS cannot necessarily be attributed to these associations.

Our analyses have some weaknesses. These include the small sample sizes particularly in small patient subgroups, which may be underrepresented in our analysis. Furthermore, TILs were assessed at one time point only, therefore excluding dynamic TIL analysis. Recent analysis suggests a particular role for sTIL measurements of residual breast cancer after neoadjuvant chemotherapy. Pelekanou et al. showed that sTIL counts showed a significant decrease after neoadjuvant chemotherapy [17]. Finally, due to the adjuvant setting for our analysis, it is difficult to distinguish a prognostic from a predictive value of sTIL analysis, since all patients in this analysis received at least one form of systemic therapy.

However, our analysis also has several strengths. First of all, we present a prospective-retrospective analysis of a randomized clinical phase III trial. Within PlanB, a central tumor bank was prospectively established to enable translational analysis at high quality and with a representative patient sample [9]. In the present analysis, we were able to estimate interobserver reproducibility, since sTIL measurements were analyzed by three independent reviewers. We found that categories were robust, with the Spearman correlation between continuous sTIL values translating into a nearly ideal association (gamma = 0.944, concordance = 87.6%) in terms of sTIL categories low, medium, and high. This is again well in line with previous analyses. Swisher et al. assessed sTIL counts among 75 samples obtained from patients with triple-negative primary breast cancer. They identified kappa statistics for sTIL evaluation of 0.57 (standard error, 0.04) for stromal sTILs and concluded an acceptable agreement in TIL count supporting the value of this biomarker for clinical use [20].

## Conclusion

In summary, our data is well in line with previous analyses demonstrating an effect of sTILs on patient prognosis in EBC. Importantly, even in intermediate early breast cancer (in association with adjuvant chemotherapy), high sTIL categories identify a subgroup of patients with a favorable prognosis. Our results underscore, that similar to HR status and Ki67, the predictive role of sTILs may be of more clinical value than their prognostic value alone.

Lastly, rather than static baseline sTIL measurement, dynamic sTIL analysis during or after neoadjuvant chemotherapy may represent a more informative tool regarding patient prognosis and treatment prediction.

## Supplementary information

**Additional file 1: Figure S1.** Design of the PlanB trial. Footnote: endocrine therapy and radiotherapy were applied according to national guidelines; E: Epirubicin; Doc: Docetaxel; C: Cyclophosphamid.

**Additional file 2: Figure S2.** Consort diagram. Percentages refer to patients in respective trial arms with follow-up data.

**Additional file 3: Figure S3.** Scatter plot of sTIL counts of Evaluator 1 vs. Evaluator 2.

**Additional file 4: Table S1.** Associations between sTIL categories and clinical / pathological variables by HR-status. sTIL categories were defined as “low sTILs” (0-10 % sTILs), “intermediate sTILs” (11-50 % sTILs) and “high sTILs” (51-100% sTILs).

## Data Availability

The datasets used and/or analyzed during the current study are available from the corresponding author on reasonable request.

## Abbreviations

DFS: Disease-free survival
EBC: Early breast cancer
EC: Epirubicin/cyclophosphamide chemotherapy
FFPE: Formalin-fixed paraffin-embedded
FISH: Fluorescence in situ hybridization
HE: Hematoxylin-eosin
HR: Hormone receptor positive
IHC: Immunohistochemistry
pcR: Pathological complete response
RS: Recurrence score
TC: Docetaxel/cyclophosphamide chemotherapy
sTILs: Stromal tumor-infiltrating lymphocytes
TMA: Tissue microarray
WSI: Whole slide image
