# Supplementary material for: Association of TILs with clinical parameters, Recurrence Score® results, and prognosis in patients with early HER2-negative breast cancer (BC)—a translational analysis of the prospective WSG PlanB trial
Source: Breast Cancer Res. 2020 May 14;22:47. doi: 10.1186/s13058-020-01283-w (PMC7227091; doi:10.1186/s13058-020-01283-w)
Supplement: Supplementary file 1 — Additional file 1: Figure S1. Design of the PlanB trial. Footnote: endocrine therapy and radiotherapy were applied according to national guidelines; E: Epirubicin; Doc: Docetaxel; C: Cyclophosphamid. [file 13058_2020_1283_MOESM1_ESM.pdf]

Supp Figure 1

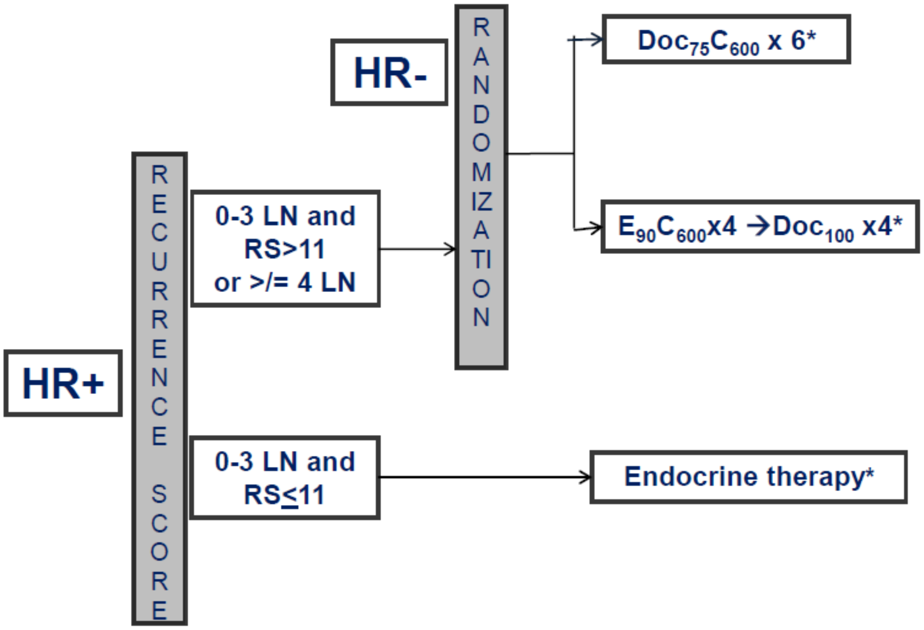

- endocrine therapy and RT according to national guidelines
- E: Epirubcin; Doc: Docetaxel; C: Cyclophosphamid
