# Supplementary figures and images for: Association of TILs with clinical parameters, Recurrence Score® results, and prognosis in patients with early HER2-negative breast cancer (BC)—a translational analysis of the prospective WSG PlanB trial
Source: Breast Cancer Res. 2020 May 14;22:47. doi: 10.1186/s13058-020-01283-w (PMC7227091; doi:10.1186/s13058-020-01283-w)

Supp Figure 2

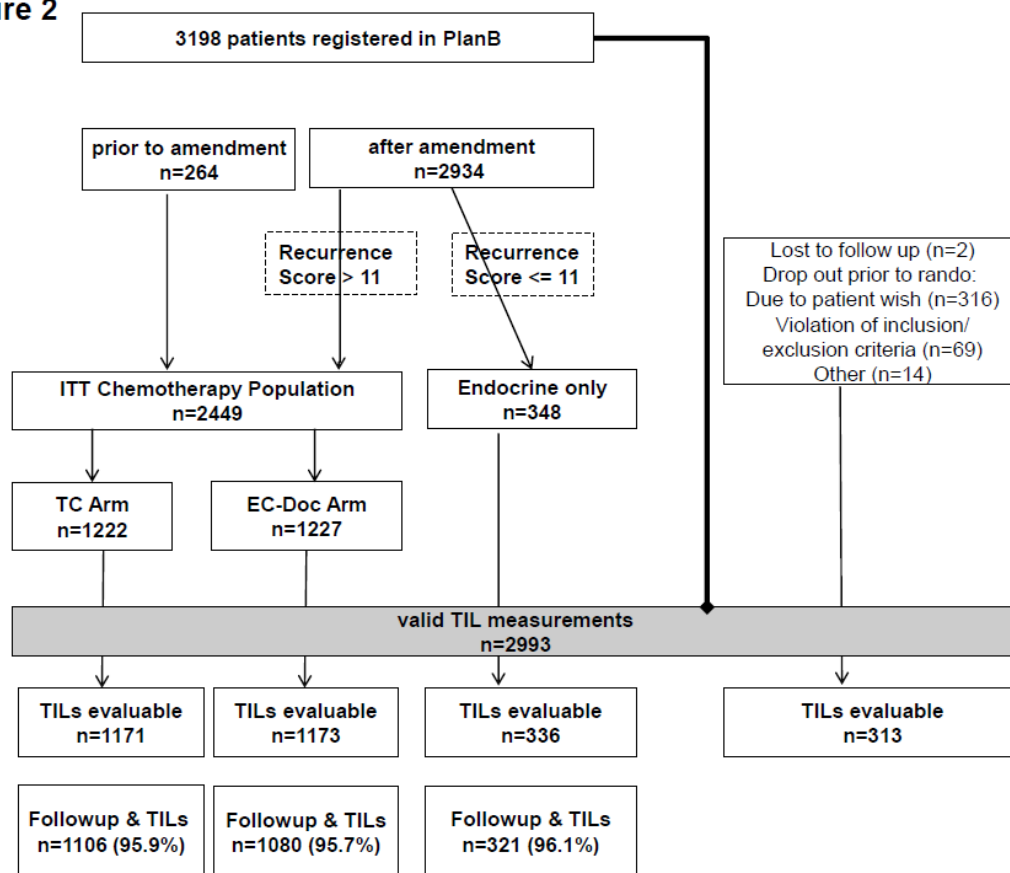

Supplement: Supplementary file 2 — Additional file 2: Figure S2. Consort diagram. Percentages refer to patients in respective trial arms with follow-up data. [file 13058_2020_1283_MOESM2_ESM.pdf]

Supp Figure 3

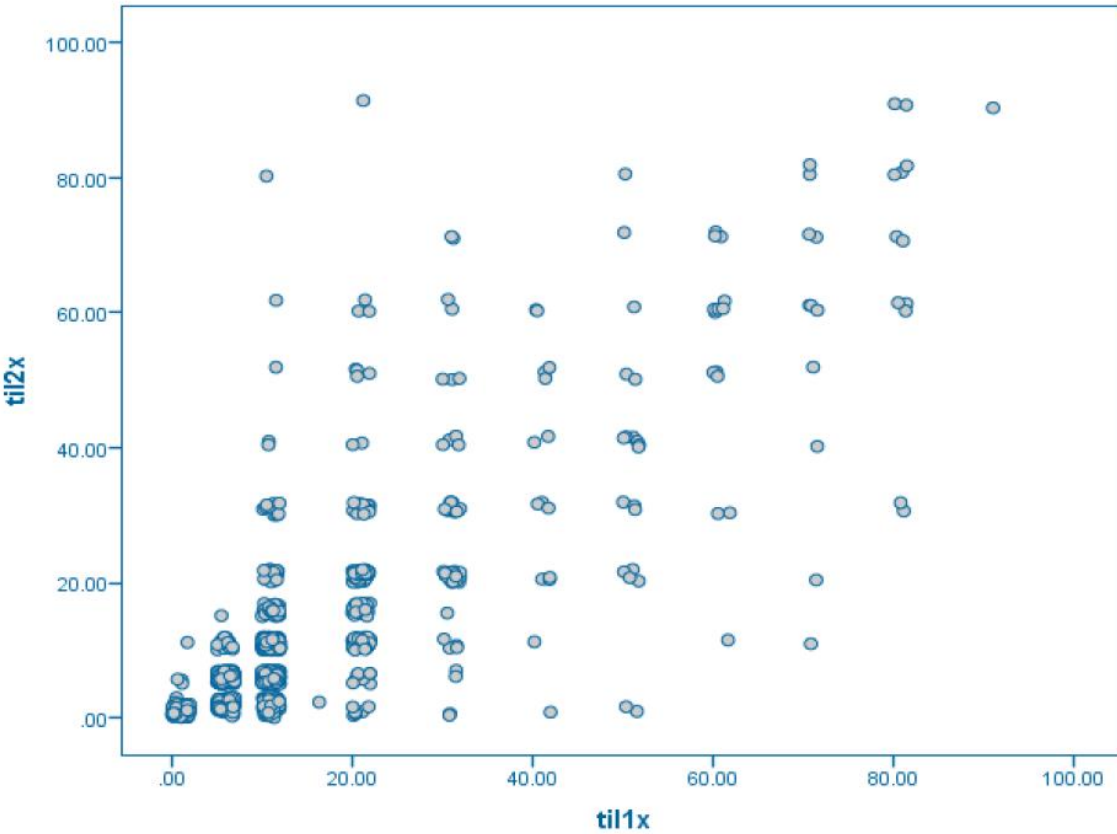

Supplement: Supplementary file 3 — Additional file 3: Figure S3. Scatter plot of sTIL counts of Evaluator 1 vs. Evaluator 2. [file 13058_2020_1283_MOESM3_ESM.pdf]
