# Supplementary material for: Association of TILs with clinical parameters, Recurrence Score® results, and prognosis in patients with early HER2-negative breast cancer (BC)—a translational analysis of the prospective WSG PlanB trial
Source: Breast Cancer Res. 2020 May 14;22:47. doi: 10.1186/s13058-020-01283-w (PMC7227091; doi:10.1186/s13058-020-01283-w)
Supplement: Supplementary file 4 — Additional file 4: Table S1. Associations between sTIL categories and clinical / pathological variables by HR-status. sTIL categories were defined as “low sTILs” (0-10 % sTILs), “intermediate sTILs” (11-50 % sTILs) and “high sTILs” (51-100% sTILs). [file 13058_2020_1283_MOESM4_ESM.docx]

**Supplementary Table 1:** Associations between sTIL categories and clinical / pathological variables by HR-status. sTIL categories were defined as “low sTILs (0-10 % sTILs), “intermediate sTILs” (11-50 % sTILs) and “high sTILs (51-100% sTILs).

|  | **HR-positive** | | | | | | **HR-negative** | | | | | |
| --- | --- | --- | --- | --- | --- | --- | --- | --- | --- | --- | --- | --- |
|  | **low sTILs**  **(valid %)** | **intermediate sTILs**  **(valid %)** | **high sTILs**  **(valid %)** | **all** | **p-value**  **(Chi Square)**  **(univariate)** | **low sTILs**  **(valid %)** | | **intermediate sTILs**  **(valid %)** | **high sTILs**  **(valid %)** | **all** | **p-value**  **(Chi Square)**  **(univariate)** |  |
| **pN** | | | | | | | | | | | |  |
| **0** | 1309 | 167 | 31 | 1507 | .643 | 190 | | 107 | 39 | 336 | .54 |  |
|  | (86.9) | (11.1) | (2.1) |  |  | (56.6) | | (31.8) | (11.6) |  |  |  |
| **1** | 789 | 106 | 14 | 909 |  | 40 | | 24 | 5 | 69 |  |  |
|  | (86.8) | (11.7) | (1.5) |  |  | (58.0) | | (34.8) | (7.2) |  |  |  |
| **2-3** | 137 | 20 | 1 | 158 |  | 6 | | 7 | 1 | 14 |  |  |
|  | (86.7) | (12.7) | (0.6) |  |  | (42.9) | | (50.0) | (7.1) |  |  |  |
| **All** | 2235 | 293 | 46 | 2574 |  | 236 | | 138 | 45 | 419 |  |  |
|  | (86.8) | (11.4) | (1.8) |  |  | (56.3) | | (32.9) | (10.7) |  |  |  |
| **pT** | | | | | | | | | | | |  |
| **1** | 1086 | 140 | 21 | 1247 | .698 | 146 | | 78 | 28 | 252 | .913 |  |
|  | (87.1) | (11.2) | (1.7) |  |  | (57.9) | | (31.0) | (11.1) |  |  |  |
| **2** | 802 | 107 | 22 | 931 |  | 83 | | 56 | 16 | 155 |  |  |
|  | (86.1) | (11.5) | (2.4) |  |  | (53.5) | | (36.1) | (10.3) |  |  |  |
| **3** | 71 | 10 | 0 | 81 |  | 4 | | 3 | 1 | 8 |  |  |
|  | (87.7) | (12.3) | (0.0) |  |  | (50.5) | | (37.5) | (12.5) |  |  |  |
| **4** | 15 | 1 | 0 | 16 |  | 1 | | 0 | 0 | 1 |  |  |
|  | (93.8) | (6.3) | (0.0) | 0.7% |  | (100.0) | | (0.0) | (0.0) |  |  |  |
| **all** | 1974 | 258 | 43 | 2275 |  | 234 | | 137 | 45 | 416 |  |  |
|  | (86.8) | (11.3) | (1.9) |  |  | (56.3) | | (32.9) | (10.8) |  |  |  |
| **age at registration** | | | | | | | | | | | |  |
| **<=50** | 718 | 109 | 17 | 844 | .183 | 77 | | 54 | 10 | 140 | .095 |  |
|  | (85.1) | (12.9) | (2.0) |  |  | (54.3) | | (38.6) | (7.1) |  |  |  |
| **>50** | 1517 | 184 | 29 | 1730 |  | 160 | | 84 | 35 | 279 |  |  |
|  | (87.7) | (10.6) | (1.7) |  |  | (57.3) | | (30.1) | (12.5) |  |  |  |
| **all** | 2235 | 293 | 46 | 2574 |  | 236 | | 138 | 45 | 419 |  |  |
|  | (86.8) | (11.4) | (1.8) |  |  | (56.3) | | (32.9) | (10.7) |  |  |  |
| **Ki67** | | | | | | | | | | | |  |
| **0-15** | 940 | 72 | 5 | 1017 | <.001 | 19 | | 4 | 0 | 23 | <.001 |  |
|  | (92.4) | (7.1) | (0.5) |  |  | (82.6) | | (17.4) | (0.0) |  |  |  |
| **15-34** | 1006 | 176 | 29 | 1211 |  | 107 | | 47 | 18 | 172 |  |  |
|  | (83.1) | (14.5) | (2.4) |  |  | (62.2) | | (27.3) | (10.5) |  |  |  |
| **35-100** | 67 | 28 | 9 | 104 |  | 92 | | 84 | 25 | 201 |  |  |
|  | (64.4) | (26.9) | (8.7) |  |  | (45.8) | | (41.8) | (12.4) |  |  |  |
| **all** | 2013 | 276 | 43 | 2332 |  | 218 | | 135 | 43 | 396 |  |  |
|  | (86.3) | (11.8) | (1.8) |  |  | (55.1) | | (34.1) | (10.9) |  |  |  |
| **Recurrence Score (high med low)** | | | | | | | | | | | |  |
| **Low**  **(0-11)** | 423 | 25 | 1 | 449 | <.001 | 0 | | 0 | 0 | 0 | .103 |  |
|  | (94.2) | (5.6) | (0.2) |  |  | - | | - | - | - |  |  |
| **Intermediate**  **(12-25)** | 13456 | 145 | 17 | 1507 |  | 0 | | 1 | 1 | 2 |  |  |
|  | (89.3) | (9.6) | (1.1) |  |  | (0.0) | | (50.0) | (50.0) |  |  |  |
| **High**  **(26-99)** | 403 | 111 | 27 | 541 |  | 4 | | 1 | 0 | 5 |  |  |
|  | (74.5) | (20.5) | (5.0) |  |  | (80.0) | | (20.0) | (0.0) |  |  |  |
| **all** | 2171 | 281 | 45 | 2497 |  | 4 | | 2 | 1 | 7 |  |  |
|  | (86.9) | (11.3) | (1.8) |  |  | (57.1) | | (28.6) | (14.3) |  |  |  |

|  |  |
| --- | --- |
